# Human movement data for malaria control and elimination strategic planning

**DOI:** 10.1186/1475-2875-11-205

**Published:** 2012-06-18

**Authors:** Deepa K Pindolia, Andres J Garcia, Amy Wesolowski, David L Smith, Caroline O Buckee, Abdisalan M Noor, Robert W Snow, Andrew J Tatem

**Affiliations:** 1 Emerging Pathogens Institute, University of Florida, Gainesville, USA; 2 Department of Geography, University of Florida, Gainesville, USA; 3 Malaria Public Health & Epidemiology Group, Centre of Geographic Medicine, KEMRI-Wellcome Trust-University of Oxford Collaborative Programme, Nairobi, Kenya; 4 Department of Engineering and Public Policy, Carnegie Mellon University, Pittsburgh, USA; 5 Department of Biology, University of Florida, Gainesville, USA; 6 Center for Disease Dynamics, Economics & policy, Resources for the Future, Washington DC, USA; 7 Fogarty International Center, National Institutes of Health, Bethesda, USA; 8 Harvard School of Public Health, Boston, USA; 9 Centre for Tropical Medicine, Nuffield Department of Clinical Medicine, University of Oxford, Oxford, UK

## Abstract

Recent increases in funding for malaria control have led to the reduction in transmission in many malaria endemic countries, prompting the national control programmes of 36 malaria endemic countries to set elimination targets. Accounting for human population movement (HPM) in planning for control, elimination and post-elimination surveillance is important, as evidenced by previous elimination attempts that were undermined by the reintroduction of malaria through HPM. Strategic control and elimination planning, therefore, requires quantitative information on HPM patterns and the translation of these into parasite dispersion. HPM patterns and the risk of malaria vary substantially across spatial and temporal scales, demographic and socioeconomic sub-groups, and motivation for travel, so multiple data sets are likely required for quantification of movement. While existing studies based on mobile phone call record data combined with malaria transmission maps have begun to address within-country HPM patterns, other aspects remain poorly quantified despite their importance in accurately gauging malaria movement patterns and building control and detection strategies, such as cross-border HPM, demographic and socioeconomic stratification of HPM patterns, forms of transport, personal malaria protection and other factors that modify malaria risk. A wealth of data exist to aid filling these gaps, which, when combined with spatial data on transport infrastructure, traffic and malaria transmission, can answer relevant questions to guide strategic planning. This review aims to (i) discuss relevant types of HPM across spatial and temporal scales, (ii) document where datasets exist to quantify HPM, (iii) highlight where data gaps remain and (iv) briefly put forward methods for integrating these datasets in a Geographic Information System (GIS) framework for analysing and modelling human population and *Plasmodium falciparum* malaria infection movements.

## Background

The recent increase in funding for malaria control through international health initiatives, such as The Global Fund to fight AIDS, Tuberculosis and Malaria (GFATM) and the President’s Malaria Initiative (PMI) [1,2], has lead to the reduction in transmission in several *Plasmodium falciparum* and *Plasmodium vivax* malaria endemic countries [3-8]. With eradication on the global agenda [9], 36 national control programmes are strongly considering, or have already set, elimination targets for the next 10–30 years [10]. Human population movement (HPM), along with drug resistance and unsustainable funding [11], has been cited amongst the significant causes of the failure of the Global Malaria Eradication Programme forty years ago [12,13]. HPM from high to low or non malaria-endemic areas can result in imported infections, challenges to health systems and onward transmission [14].

The number of incoming malaria-infected travellers each month or each year determines the "vulnerability" of an area (defined by the risks posed by imported infections, for example through influxes of infected individuals from higher to lower transmission areas) [15,16]. Each imported case presents a risk of initiating outbreaks, epidemics, or increasing local transmission levels in areas of high "receptivity" (the historical potential for vector transmission that determines the severity of local onward transmission) [16]. Imported infections do, however, have clinical significance in low or zero receptivity areas too, if infected individuals become ill at their destination and require medical care. Together, vulnerability and receptivity form the mathematical basis of "malariogenic potential" (the overall risk that malaria could return after elimination), an important measure for all malarious areas aiming for elimination [16,17]. Understanding HPM patterns to and from high transmission regions is therefore important for designing strategic evidence-based control plans. Surveillance systems may be effective in detecting symptomatic cases, for example through hospital patient travel history records [18] and border screening [19-21], however, they are less likely to detect those not seeking treatment, including asymptomatic imported cases. Therefore, quantification of HPM is valuable when selecting appropriate control strategies and when developing a comprehensive elimination feasibility assessment - a key pre-elimination planning tool that quantifies technical, operational and financial feasibility of an elimination agenda [21,22]. Zanzibar presently remains the only example where an extensive mathematical quantification of HPM and *P. falciparum* malaria importation has been undertaken and put into context for strategic planning through a complete elimination feasibility assessment [15,21,23,24].

HPM patterns vary substantially by time scales, spatial scales, motivations for travel and socioeconomic and demographic characteristics of travellers [25,26], as does the risk of becoming infected, the type of malaria parasite, and illness caused [27-29]. Therefore, the implications of HPM on malaria can be better understood when given more precise spatial-temporal dimensions [30]. In general, the most important component for infection importation and onward transmission is HPM from low transmission, high receptivity areas to high transmission areas and back, and unidirectional HPM from high to low transmission areas [15].

*Plasmodium falciparum* parasite rate (*Pf*PR) maps that represent current transmission risk under control [31-33], population distribution maps [32,34] and mathematical models [35-38] have advanced in their sophistication and detail in the past decade and can be linked to HPM data to estimate the risks of infection acquisition in travellers, for assessing the implications of HPM on *P. falciparum* malaria transmission [15,23,39]. The mapping and modelling framework for *P. vivax* malaria is less developed, but is continuing to be improved [40,41]. Various types of data exist and have been used to quantify HPM, such as call record data to track mobile phone users' locations [42], models assessing movement trajectories [43], previous versus current residence data from population censuses [44], trips made abroad from national survey data [45] and travel histories of hospitalized patients [46]. However whilst existing studies based on mobile phone call record data combined with malaria endemicity maps have begun to quantify within-country HPM and malaria dispersion patterns [47], quantitative knowledge of cross-border HPM, demographic and socioeconomic breakdowns of HPM, seasonality of HPM, modes of transport, personal malaria protection and motivations for HPM are often poorly developed, despite the importance of these factors in accurately gauging movement patterns of possible parasite-carrying individuals.

This review describes survey and other relevant data types to illustrate the importance of HPM across different spatial and temporal scales that are relevant for *P. falciparum* malaria control and elimination assessments, and document where datasets exist to quantify these types of HPM. Previous studies that use data to make *P. falciparum* malaria-relevant conclusions on HPM patterns and highlight where data gaps remain are reviewed. Finally, methods for integrating these datasets in a Geographic Information System (GIS) and mathematical modelling framework to analyse and model human and *P. falciparum* malaria infection movements are put forward.

### Defining human population movements relevant for malaria transmission and control

**Figure 1:**
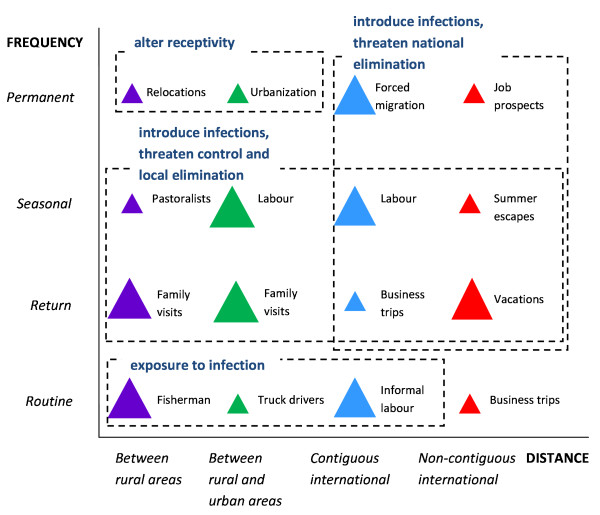
**Examples of human population movement (HPM) types relevant for malaria control and elimination; Human population movement (HPM) is stratified by spatial (distance travelled) and temporal (frequency of travel) characteristics.** The blue and red triangles represent between country HPM (non-contiguous international and contiguous international) whilst the purple and green triangles represent within country HPM (between rural areas and between rural and urban areas). The size of the triangle illustrates the importance of the HPM category for estimating malaria importation, based on infection importation risk of the individual traveller and aggregate flow of travellers in each HPM category. Table 1 and 2 provide details of data types that may be used to quantify the different HPM categories.

Stoddard *et al*[30] describe a framework for categorizing the types of HPM important for vector borne diseases across spatial and temporal scales. Here, this framework is extended by identifying data sets that can help to quantify HPM that are important for *P. falciparum* malaria control and elimination plans (Table 1, Figure 1). All HPM is roughly categorized by the sorts of data available to quantify HPM types: HPM between countries (non-contiguous international HPM and contiguous international HPM) and HPM within countries (intra-national HPM between rural and urban areas and intra-national HPM between rural and other rural areas) for four different temporal scales (permanent/long term, periodic/seasonal, return/short term and routine). Permanent or longterm HPM includes migratory movements or relocations that involve change of residence. Periodic HPM includes those that follow a yearly cycle, usually based on growing seasons, tourism trends or industrial demand. Return or short term HPM includes trips away from and back to a normal place of residence. Routine HPM includes frequent, regular travel such as daily commutes to work or school. Then, an illustration of how each HPM category may have a different implication for malaria transmission and the control agenda is provided. An assessment of the importance of demographic, socioeconomic and motivational factors, review methods used in existing studies that relate HPM to malaria and point out gaps in data availability and potential analysis tools is also given. HPM at fine temporal and spatial scales, when assessing heterogeneous exposure and transmission risk at these scales, is assessed as a separate category. Finally, a GIS and modelling framework and basic methods to analyse and model human and *P. falciparum* malaria infection movements is proposed.

**Table 1:** Retrospectively collected migration and travel history HPM data sets and sources

**Data set description**	**Malaria-relevant HPM data**	**Data sources**^**+**^
**Census data:**		
Complete/subsets of raw census data	Birth place, previous/current residence, demographic	National Statistics Bureaus
Aggregated census data	Birth place, previous/current residence	Migration matrices from published census reports
Census Microdata	Birth place, previous/current residence, demographic data	International Public Use Micro-Samples (IPUMS) [https://international.ipums.org/international/]
Bilateral migrant stocks	Foreign migrants with birth place, current residence	Development research centre on Migration, Globalization and Poverty (MigrationDRC)
		[http://www.migrationdrc.org/research/typesofmigration/ global_migrant_origin_database.html]
**Household Surveys:**	Time away in last 12 months, number of trips, reason for travel, birth place/current location, demographic	International Household survey network
National Household Budget Surveys		
		[http://www.internationalsurveynetwork.org/ home/?q=activities/catalog/surveys]
Migration and Remittances Surveys	Rural/urban classifications previous and current location, demographic	Open access: [www.developmentdata.org/hh_surveys.htm]
World Bank Living Standard Measurement Surveys (LSMS)	Time away in last 12 months, time in current location, current/previous residence, reason for move, birth place, demographic	World Bank Central Microdata Catalog [http://microdata.worldbank.org/index.php/home]
Demographic Health Surveys (DHS)	Rural/urban status of locations, demographic	[http://www.measuredhs.com/start.cfm]
Malaria Indicator Surveys (MIS)	International trips in last x months, destination, demographic	[http://www.malariasurveys.org/surveys.cfm]
Labour Force Surveys	Time in previous/current residence, reasons for move, demographic	[http://www.ilo.org/dyn/lfsurvey/lfsurvey.home]
**Small-scale and sub-population surveys**	Number of trips made away, destination, duration of stay at destination, transport used, reason for travel, demographic, socioeconomi	Development research centre on Migration, Globalization and Poverty (MigrationDRC)
e.g. school children, urban city, hospital and border survey		[http://www.migrationdrc.org/publications/resource_guides/ Migration_Nationalsurveys/child_db/home.php?function= search&empty_search_variables=1&table_name=Migration_Info]

^+^ A collection of surveys is available for open access @ Openmicrodata: www.openmicrodata.org.

### HPM between countries

**Non-contiguous international HPM.** Non-contiguous international HPM is defined here as movements between countries that do not share a border. This HPM type is most relevant to malaria transmission and control in terms of likely imported infections into low or non-endemic countries from countries with higher transmission [48]. Tourists visiting and migrants from malaria endemic countries have for many years led to imported infections into malaria-free countries [49-51]. Planned relocations between countries may also lead to imported infections, for example, relocations of Somali refugees to the United States [52]. Depending on local environmental and climatic conditions, imported infections may lead to onward transmission, epidemics and outbreaks and threaten the success of previous control or elimination efforts [14]. Non-contiguous international HPM is most relevant for countries with unstable endemic-prone malaria and with elimination as their national goal.

To understand non-contiguous international HPM as relevant for a malaria importation analysis, HPM can be further categorised by temporal characteristics. These include permanent/long term, seasonal, short term/return and routine travellers (Figure 1). Permanent/long term migration, such as movement of skilled migrants, for example doctors, nurses, lecturers, engineers, scientists and technologists from Ghana and Nigeria (higher transmission countries) to South Africa (a relatively lower transmission country) [32,53] may result in imported infections through symptomatic and asymptomatic parasite carriers. Periodic/seasonal migration of Indian labourers to the Middle East [54] threaten imported infections if an individual travels home and is susceptible to infection. Short term/return travellers (e.g. holiday-makers and people visiting family) may get infected upon travel to higher transmission zones and return with parasites in their blood. Routine travellers, such as business air passengers, are unlikely category of non-contiguous international HPM type to import infections, as they may sleep under bed nets and in air conditioned rooms and therefore have minimal exposure to infection.

Health system surveillance databases may be used to identify the sources of imported infection, for example, through an assessment of patient travel history records to identify origins of imported infections and/or through active surveillance by testing in-coming travellers for infection at gateways into countries, as previously carried out at international airports in Oman [55]. Testing at entry points for the case of Mauritius, however, was recently shown to provide marginal benefits for large investments [20]. Furthermore, targeted screening, for example to fever-presenting individuals, may miss asymptomatic parasite carriers. Therefore, to quantify non-contiguous international HPM, various other data sources can be used (Table 1). For example, previous residence and birthplace records from South African census data can be used to estimate numbers of resident Ghanaians and Nigerians, and specifically, with freely available census microdata [56], traveller demographics may help determine reasons for relocation. Periodic and seasonal movement however is more difficult to estimate from census records, but may be estimated from small-scale or sub-population surveys [57]. Some national household surveys such as the Malaria Indicator Surveys (MIS), contain travel history data that may allow estimation of short-term international travel (e.g. Djiboutian travellers to Ethiopia, Yemen, Eritrea, Somalia, Mozambique and Saudi Arabia [45]). Routine non-contiguous international movements are less common and usually confined to airline passengers. Detailed flight passenger data is less readily available, however. Flight schedule data (Table 2) that indicates high volume routes may be used as a proxy, but it is not generally possible to extrapolate and directly assess the characteristics and origins/destinations of individual travellers – for example, transit passengers cannot be differentiated, and demographics and motivations are unknown.

**Table 2:** Passenger flows and transport route data to illustrate connectivity between locations

**Routes and flows**	**Data set description**	**Connectivity**	**Data sources**
Flights	International and domestic passenger flows	Monthly passengers per route	National Airport Authorities
			OAG Worldwide Ltd [www.oagaviation.com]
		Monthly incoming and outgoing flights in and out of principle international airports	
	International flights scheduled		
Shipping/ trade routes	Stations served and transport network	Most visited seaports and routes in 2000	[http://www.infoline.isl.org/index.php? module=Pagesetter&func=viewpub&tid=4&pid=3]
			[http://www.nceas.ucsb.edu/globalmarine/impacts]
			
Bus routes	Stations served, transport network and schedules	Inter-city routes	Public and private bus companies
Roads	Road networks	Internal and cross-border routes	Trans-Africa Roads network
			[http://www.ciesin.columbia.edu/confluence/ display/roads/Global+Roads+Datah]
		Within country routes: major roads, road type and quality, annual average traffic	
	Road traffic and characteristics e.g. quality and size		Compiled collection of surveys e.g. [http://www.krb.go.ke/index.php/road-conditions]
Railway	Stations served, transport network and schedules	Cross-border routes	Online Rail Service Information [http://www.railserve.com/Passenger/Africa/]
		Internal commuter routes	Railways Corporations
Ferry	Ferry schedules	e.g. between Tanzania mainland and Zanzibar	Zanzibar Ministry of Communication and Transport [http://zanzibarmagic.com/english2nd/zanzibar-dar- es-salaam-tanzania railway bus ferry speed boat schedule taxi fares.htm e.g. http://azammarine.com/]

Some of these data have been used previously to assess the implications of non-contiguous international HPM on malaria movements. The importance of each type of HPM and nature of the data determines how data can be linked with spatial malaria data. For example, census derived bilateral migrant stock data has been used to assess the implications of population exchange between countries on malaria transmission and elimination strategies at a global scale. Tatem *et al*[58] combined HPM data and a global *P. falciparum* malaria endemicity map [31] to map communities of countries with relatively high infection exchange, defining "natural" malaria movement regions. Global flight and shipping route data have been used to assess dispersal of vectors [59-61]. Some types of non-contiguous international HPM relevant for malaria, such as periodic or seasonal migration between countries and short term return movement between countries, however are more difficult to quantify and relevant data are less accessible.

**Contiguous international HPM.** Contiguous international HPM (or cross-border HPM) is defined here as that between countries sharing national borders and usually connected by road or ferry networks. Contiguous international HPM has been attributed to maintaining high transmission hotspots at border points [62] and imported infections that threaten elimination success [58]. For example, imported infections from Yemen into Saudi Arabia continue to challenge Saudi elimination efforts [51]. Similarly, imported infections from Angola into Namibia challenge Namibia’s 2020 elimination target [63].

**Figure 2:**
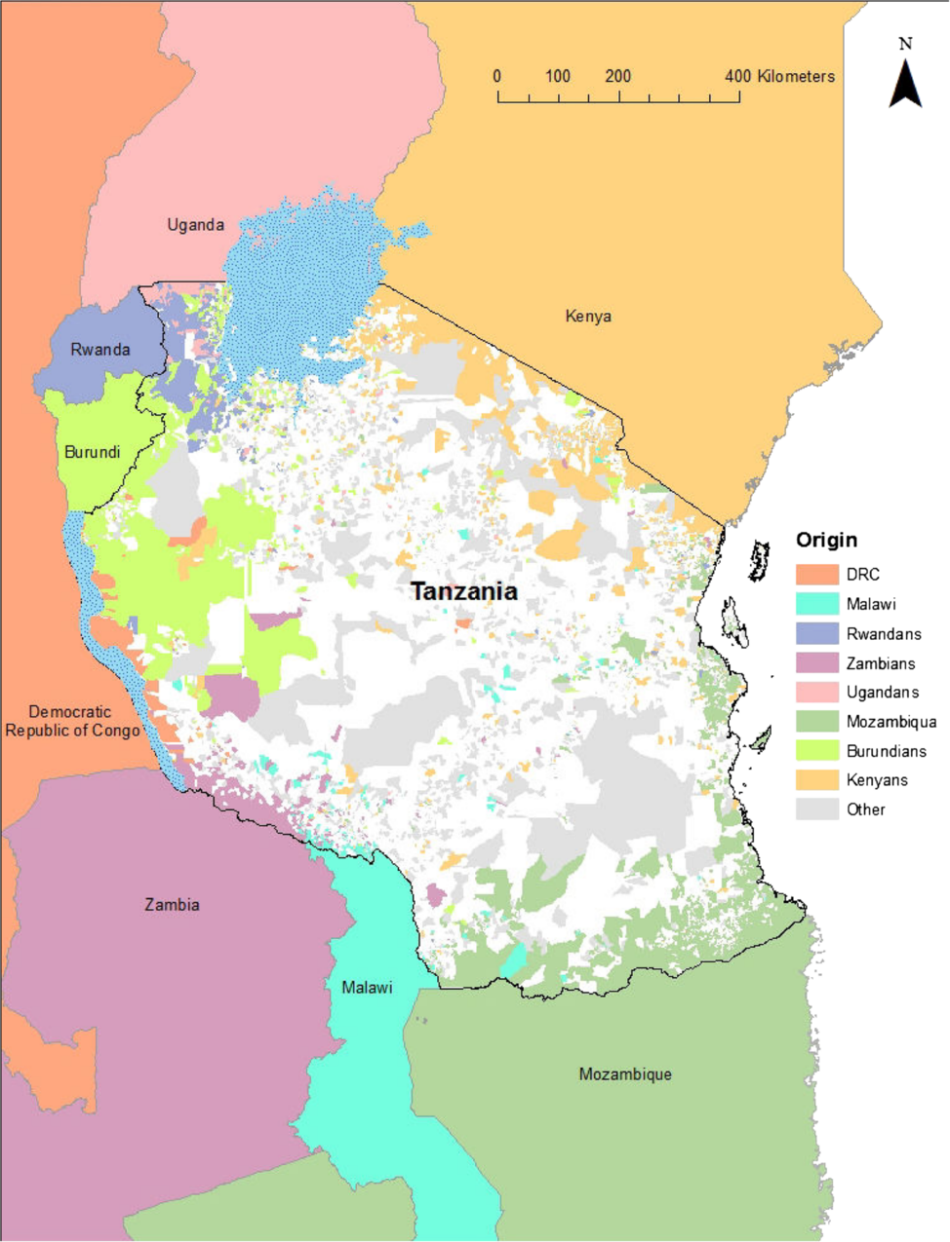
**Census data records showing place of birth of population enumerated in the Tanzania 2002 census; Resolution of current location of individuals was recorded at a ward level, whilst place of birth was recorded at a country level, representing origin of current Tanzanian residents.** Wards are colour-coded according to place of birth of majority of non-Tanzanian enumerated individuals.

Contiguous international HPM spanning different temporal scales have different implications on malaria transmission and control. Temporally categorised cross-border HPM includes permanent/long term, seasonal, short term/return and routine travellers (Figure 1). Permanent migrants may consist of ex-refugees (e.g. populations born in Burundi now residing in Tanzania (Figure 2)) and other forced migrants in Eastern Africa [64], and displaced populations in border camps in Thailand [65] illustrate permanent/long term cross-border movement. Movements of labourers from various countries in Southern Africa into South Africa [53] (similarly, Angolan labourers in Namibia [57], Yemeni labourers in Saudi Arabia [66]) and Haitian labourers in the Dominican Republic [67] illustrate seasonal cross-border HPM. Short term/return cross-border HPM include Afghans crossing borders into Pakistan for immediate income [68], cross-border return trips by those claiming to be refugees but often travelling to/from home country and return cross-border HPM for purposes such as shopping as seen at the border of Angola and Namibia [57]. Routine travel, such as Djiboutian fisherman regularly embarking at Eritrean and Yemeni shores and Angolans frequently crossing the Namibian border to shop or visit family [57], also constitutes cross-border malaria-relevant HPM.

Various datasets (Table 1), similar to those assessing international non-contiguous HPM, can be used to assess cross-border travel. Foreign-born migrants can be estimated from census data to assess long term/permanent cross-border HPM, for example, comparing current residence to birthplace for individuals enumerated in Tanzania's 2002 census (Figure 2). Foreign-born migrant data may also be used as a proxy for short temporal scale travel, as people go back and forth visiting relatives left behind. Short term return travel from low to high and back to low transmission areas generally has a larger significance for onward transmission in home regions due to higher risk of infection (lack of immunity and longer duration of stay in low transmission area [15]). Border crossing points and cross-border surveys can provide information on seasonal patterns of movement, however, this data is more suitable for estimating short-term/return travel, as detailed data may only be available for a year, and it is often not archived [57]. Railway and bus passenger traffic data may be used to record flows between adjacent countries (Table 2), providing proxies for cross-border travel, however, routine cross-border travel may go unrecorded in developing countries as much of it occurs informally. For example, routine cross-border HPM where individuals travel short distances between neighbouring countries for work/shopping purposes are difficult to track [57].

Some cross-border HPM data has previously been used to assess the implications of cross-border HPM on spatial patterns of malaria, for example, national malaria indicator surveys in refugee populations that record travel history [45], but generally, compared to other HPM types, detailed cross-border malaria-relevant HPM assessments have rarely been made. Compared to non-contiguous international HPM however, contiguous international HPM occurs more frequently and in larger volumes and therefore may be of substantial concern for countries aiming for elimination. High-quality datasets that inform on such movements are required, however, they are often difficult to obtain. The Angolan-Namibia border only artificially separates people that are ethically homogeneous and move regularly across the border, making it unrealistic to consider that the populations on the two sides of the border are different. However, HPM datasets and malaria control programs usually focus just on single country datasets. For example, analysis of datasets such as mobile phone call record data [47] is difficult to do in these situations because of practical limitations on subscriber identity module (SIM) cards and network operator coverage. Similarly, detailed travel history surveys may be focused on a sample population on only one side of an “artificial” border [57].

### HPM within countries

**Figure 3:**
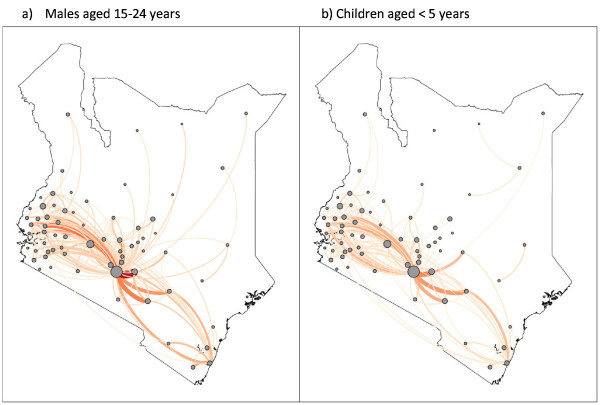
**Comparing HPM in different demographics from Kenya 1999 census microdata; HPM between two district locations recorded if previous residence differs from current residence.** Circles at the centre of the district represent locations and circle size is proportional to the population size of the district. Flows between locations are represented by a line between two circles and line width is proportional to the number of people that move between two locations. **a)** HPM flows in the male population between the ages of 15 and 24 years, **b)** HPM flows in children under the age of 5 years. Origin–destination pairs with less than 10 HPM flows were omitted from illustration.

**Intra-national HPM between rural and urban areas.** Urban areas offer employment opportunities, resulting in large rural to urban HPM in many developing countries, particularly of young adults (Figure 3) [69,70]. Many urban migrants are likely to maintain family ties with their rural homes, resulting in connectivity between rural and urban areas and travel back and forth [70], which can result in within-country infection importations to malaria-free districts, as seen in urban South African districts [71]. Intra-national movements are likely relevant to malaria epidemiology, as urban areas often have lower transmission [72-74]. Infected rural travellers may import infections into urban areas. Urban dwellers may acquire infection upon visiting rural homes and bring infection back to urban locations [73]. Quantifying HPM between rural and urban areas therefore assists in understanding urban malaria, for example in Nairobi, Kenya, where imported infections may risk onward transmission [75].

Internal HPM between rural and urban areas mostly consists of permanent or long-term rural–urban migration, especially in developing countries. However, other temporally categorised types of internal HPM between rural and urban areas (Figure 1) include labour-related seasonal HPM [76], return travel by urban business owners residing in rural areas that make short trips to urban areas to stock shops for example, and daily movements of rural residents to work and schools in urban areas (daily activities, such as working on construction sites where stagnant pools of water and mosquito breeding are likely, determine individual’s risk of exposure to infection. HPM at fine temporal and spatial scales are covered in a separate section).

**Figure 4:**
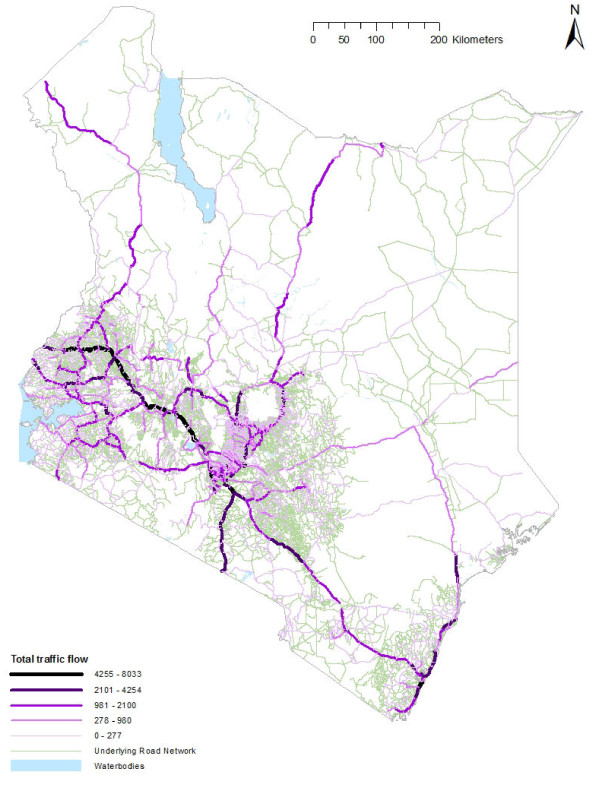
**A road network map of Kenya with road traffic data shown for sub-sections of the roads;** Green: all roads with no traffic data, darker colour represents areas with large traffic volumes. Table 2 provides details of data collection and compilation.

Data useful for quantifying permanent internal migration is commonly recorded (Table 1). Census data, addressing birthplace and previous residence (e.g. place of residence one year ago) of enumerated individuals are the most common measure of permanent migration (Figure 2 and 3 show HPM records from census data). Regularly collected household surveys also collect birth place data and may provide a proxy for shorter-term HPM, for example, Ghana’s Living Standards Survey which collects detailed migration and demographic data for individuals enumerated [77]. Regularly conducted surveys such as Demographic Health Surveys address rural/urban descriptions of previous and current residence, a proxy for rural–urban HPM. Short term HPM between rural and urban areas is often recorded in a variety of travel history surveys [45,78]. This type of data may also provide indications for seasonal labour-related HPM, depending on the questionnaire structure and content, such as time and duration of trips. Routine/daily HPM may also be estimated using small-scale travel history surveys, recording number of trips, distance and transport used for commutes to and from work or school. Road traffic and railway passenger data may provide proxies for assessing flows in and out of urban centres (Table 2, Figure 4). However, data on start and end points of individual travellers and other personal details, such as demographic features and access to prophylaxis, are not readily available.

**Intra-national HPM between rural areas.** Internal movements between rural areas have implications for malaria transmission and control. For example, population relocations between rural areas may establish new disease foci [79]. Rural to rural HPM may also have implications for populations at risk of infection, for example, Uganda internally displaced person (IDP) camp residents, where women are more likely to be infected than men, as men are likely to travel out of camps to highland areas where transmission is lower [80]. This HPM type plays a role in maintaining connectivity, in terms of infection exchange, between different transmission zones. It is most relevant for countries with overall low transmission but higher transmission hot spots in certain areas.

Defining internal HPM between rural areas in temporal categories include: permanent or long term HPM, such as population relocations (displaced populations from volatile areas to rural refugee camps may travel from higher to lower transmission areas and threaten imported infections), seasonal labour HPM of plantation workers [81,82] (labourers from high transmission areas carry parasites in their blood that may be transmitted at destination), short term/return HPM between rural areas may include visits to family and friends (individuals infected upon travel may return with parasites in their blood and threaten onward transmission in high receptivity areas), and routine HPM such as travel to work or school in nearby villages (HPM at fine temporal and spatial scales are covered in a separate section below).

Methods and data for quantifying movements between rural areas are similar to those described for internal HPM between rural and urban areas however they may be more likely to go unrecorded. For example, bus passengers travelling between different rural areas are likely to have layovers in urban centres and as individual tickets may be issued, the rural to rural trip may not be detected in inter-city routes data (Table 2).

### HPM at fine temporal and spatial scales

In addition to the demographic and socioeconomic characteristics and bednet use of an individual traveller, the highly heterogeneous local nature of individual exposure and transmission risk determine an individual’s probability of infection. For example, an individual’s risk of exposure to infection varies depending on the proximity of their home to mosquito larval sites and how long a person spends in this proximity during mosquito biting hours [30]. Therefore, there is a need for high resolution spatial and temporal HPM data if an understanding of small-scale heterogeneity in transmission is to be developed.

There are various types of high-resolution data collection and analysis methods, such as GPS data-loggers and individual mobile phone records (Table 1 and as described in Stoddard *et al*[30] in more detail) [30,42,83], as well as mathematical techniques [84] used to quantify HPM at fine scales. GPS data has previously been collected to quantify HPM as relevant for vector borne diseases [83]. Mobile phone usage data may also be useful for studying HPM at this scale, though is constrained by the distribution of receiving towers [42]. These studies remain few, expensive and most relevant to a few specific areas of interest. However, study methods may be adapted to obtain useful data in other areas. HPM data at fine resolutions may also be used to parameterize individual-based transmission models, which may allow imported infections to be estimated in areas where data is not available.

### Geographical Information System (GIS) and modelling framework

Spatially located origins and destinations of travellers and HPM flows, together with transport network data (Table 2), spatial disease risk data (*eg* endemicity maps [85]), population distribution data [86] and mathematical modelling techniques can be combined within a GIS modelling framework to allow quantitative analysis of HPM and estimated malaria infection movement in a common platform.

**Figure 5:**
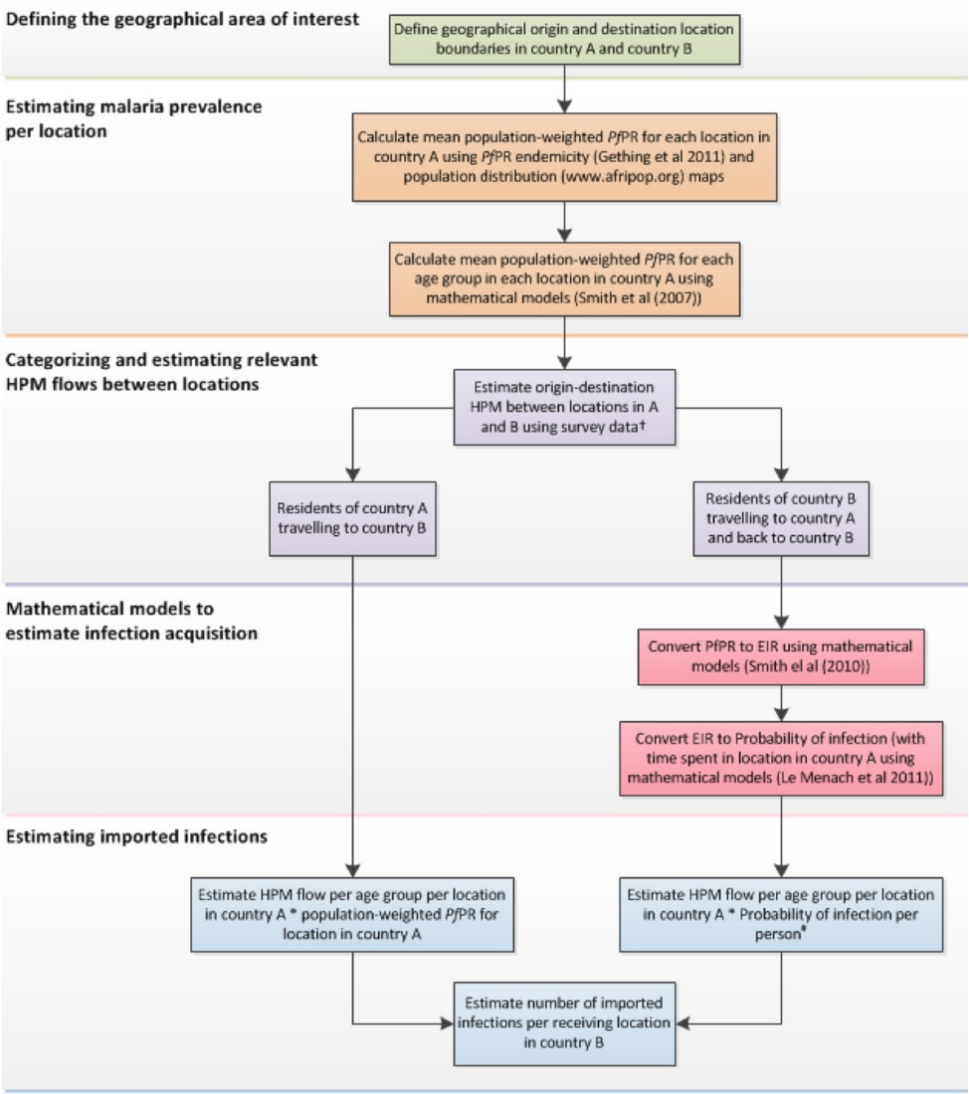
**Steps to estimate the impact of contiguous human population movement (HPM) on malaria importation; Steps to estimate the impact of contiguous human population movement (HPM) between country A and country B (country A with relatively higher transmission than country B), on*Plasmodium falciparum*(*Pf*) malaria importation in country B, using Geographical Information System (GIS) tools and mathematical modelling techniques.**

Within a GIS, layers of different spatial information can be input and overlaid to obtain geographically specific, disease-relevant outputs and combining these with mathematical models can yield importation-relevant measures, such as the number of imported infections per 1,000 of the population per year [16]. Figure 5 illustrates an example of a *P. falciparum* malaria-relevant HPM analysis of cross-border HPM data between country A and country B (country A has relatively higher transmission than country B), based on models previously outlined [15,87,88]. Pre-defined geographical boundaries can be overlaid onto *Pf*PR endemicity [32] and population distribution maps [86] to estimate population-weighted *Pf*PR per location. Using mathematical models, the population-weighted *Pf*PR can be further stratified by age to obtain age-specific population-weighted *Pf*PR per location [89]. Using travel history records from cross-border survey data (Table 1), the directional HPM flows between locations can be estimated. Methods to estimate infection acquisition vary for different traveller groups [15]. Therefore, HPM flows may be divided into two broad categories: residents of country A travelling to country B and residents of country B travelling to country A and back. For residents of country A travelling to country B, HPM flows may be weighted according to origin location population-weighted *Pf*PR. For residents of country B travelling to country A and back, data on estimated time spent in locations in country A (relatively higher transmission area) and mathematical models [15,36] may be used to estimate the entomological inoculation rate (EIR), the probability of infection acquisition per person and the number of imported infections per origin location. By overlaying this on a population distribution map, the number of imported infections per 1000 of the population in each administrative unit in country B can be estimated. If HPM can be age-stratified, age-specific population-weighted *Pf*PR per location, and similar infection acquisition methods, may be used to assess the demographics of imported infections. Additionally, seasonal malaria transmission maps can be used to assess months in which imported infections per origin location are most likely [23]. As with most HPM data and mathematical models this exercise involves uncertainties, such as uncertainty from estimating HPM using individual recall data (travel history data) or incomplete mobile phone call record data, uncertainty from constructing *Pf*PR maps [90], uncertainties from using *Pf*PR as a measure of endemicity in low transmission areas [91] and uncertainties from lack of data on individual’s use of prophylaxis and bednet use. Despite the uncertainties, these models can be one of the most effective ways of synthesizing available data and making useful recommendations about the relevance of HPM for malaria control planning.

## Discussion

HPM, specifically in low transmission and elimination settings, is important to estimate for the success of control and elimination programmes. As the failure of previous control efforts shows, neglecting HPM from high to low transmission areas, can lead to re-emergence in areas where low transmission had been previously achieved but receptivity to infection remained high [27,92]. Countries aiming for elimination have, therefore, been advised by the WHO to carry out elimination feasibility assessments [93], which encourages the use of evidence-based methods to estimate infection importation risk. Among the various types of HPM illustrated in Figure 1, those with certain spatial and temporal characteristics and specific demographic and socioeconomic characteristics may be more likely to travel and import infections. As Figure 3 shows for Kenya, males between the ages of 15 and 24 years are more likely to migrate (assessed according to current and previous location of residence) than children in the same population. According to previous *P. falciparum* malaria-relevant HPM analyses done in Zanzibar [15], susceptible residents travelling from the low transmission environment of Zanzibar to higher transmission areas (mostly in mainland Tanzania) and returning with infections may be more likely to result in onward transmission in Zanzibar compared to Tanzanian residents travelling to Zanzibar, primarily due to the length of stay in high transmission location and duration of infectious period spent in Zanzibar [23]. *Plasmodium falciparum*-infected individuals travelling to low transmission, high receptivity areas pose a larger concern for elimination programmes than infected travellers moving to high transmission, high receptivity areas or low transmission, low receptivity areas. Furthermore, as national surveillance systems may detect symptomatic imported cases, asymptomatic parasite carriers and infected non-healthcare seekers are likely to go undetected unless identified through active case-detection or individually screened at entry points [94]. Large-scale screening can be a significant expense and it would be financially difficult for most malaria elimination regions to sustain [20]. However, identifying, testing and treating high-risk traveller groups that could potentially be targeted for specific preventive control measures, such as sugarcane plantation migrant workers in Swaziland from Mozambique [95], may be more cost-effective. Quantitative understanding of the details of HPM patterns is useful for assessing elimination feasibility and feeding into models that can assess the operational and financial burdens of different strategies. Making precise HPM quantifications to obtain malaria-relevant details, especially where data is scarce, poses a challenge to feasibility assessment projects. However there are plenty of dispersed datasets (Tables 1 and 2), which if carefully examined, can provide a starting point for further malaria-relevant HPM investigations (Figure 5).

The datasets discussed here illustrate the HPM types that can be quantified (Table 1) and where gaps may exist. Figure 2 provides an indication of the likely origins of cross-border migrants, indicating the need for more detailed quantitative information for short-term cross-border movement, as migrants likely to visit their origin countries in the future. The existing data do however cover a large variety of HPM patterns. In general, census data provides useful information on long term HPM, indications of family ties that drive short-term visits, and demographic characteristics for national populations. Census data can also be used to assess population composition (Figure 3), useful when devising infection detection and control methods, as risk of infection differ between different demographic groups [89]. Household surveys provide data to address both long-term and short-term movements for nationwide samples, providing details on types of travel, such as family visits and vacations. Other surveys that focus on smaller geographic areas and specific sub-populations, for example border point surveys, may provide even more detailed HPM data than nationwide surveys, such as reason for travel and mode of transport used [96]. Routine HPM data remain rare. High spatial and temporal resolution HPM data collection methods are generally expensive, but may be effectively used to capture routine HPM.

Considering the various constraints on individual datasets, using multiple, complementary datasets (Tables 1 and 2) allows for a more detailed understanding of HPM. However, some data gaps will likely remain unfilled. For example, duration of stay in high transmission areas is amongst the more important malaria-relevant HPM metrics, but is rarely available from census and survey data. Similarly, other gaps in survey and census data include travellers' use of prophylaxis, place of stay upon travel and activities engaged in upon travel. Additionally, some types of HPM are more readily quantifiable from the data available compared to others. Routine international and cross-border HPM are difficult to quantify from existing data. Some HPM data, such as previous trips records from household survey data (Table 1), may provide an indication of HPM seasonality (using time of most recent move records). However, quantifying precise seasonal inferences is a challenge. Various surveys record the number of trips made in last 12 months or time spent away in the last 12 months (Table 1), however they do not give an indication of locations visited, providing an incomplete platform for assessing malaria-relevant HPM. Some malaria-relevant HPM may also go unrecorded, for example the large influxes of refugees, internally displaced people and illegal immigrants who do not disclose cross-border relocation [97]. Finally, datasets differ from place to place and household surveys done in one country may not adequately capture relevant movements elsewhere, or be undertaken with the same set of questions. Adding questions to existing surveys, on such aspects as place and duration of stay in visited locations, travellers’ use of bednets and prophylaxis, malaria episodes and activities during travel that may increase risk of infection, e.g. farming, would improve the utility of survey HPM data in estimating infection acquistion. Moreover, standardizing such survey questions between different locations would allow for more rigorous between-country comparisons. Recording travel patterns over time using longitudinal study designs, may also enable seasonal HPM inferences from survey data.

Recently, mobile phone usage data have been used to capture nationally comprehensive, high spatial-temporal resolution, individual-level data on within country HPM and link it to disease data [98]. However, although individual call volumes could be used as a proxy for the socio-economic status of phone-users, mobile phone usage data do not directly capture demographic and socio-economic descriptions. The potential exists though to combine such data with demographic descriptions available from surveys, providing valuable detail on the demographics of HPM. Additionally, high resolution HPM information from mobile phone usage could potentially be used to parameterize HPM models. For example, directional HPM data and distance estimates (e.g. road distances and approximate travel time obtained using road networks in the GIS framework) between locations may be used to parameterize gravity-like models [99], and demographic stratifications of directional HPM may then be used to develop more detailed gravity models. The high resolution HPM data, such as travellers’ duration of stay in high transmission locations, may be combined with existing transmission models [36], prevalence maps [32,85] and population distribution maps [86] to quantify imported *P. falciparum* infections (Figure 5). Modelling may then be used as tool to overcome uncertainties where HPM data does not exist [88] and inform policy makers, within the bounds of uncertainty, on how to mostly effectively invest in control or elimination plans. Furthermore, beyond the survey data that exists, new approaches to collect detailed malaria-relevant HPM data may be explored. For example, incorporating additional questions on travel history in existing data collection systems [100], as described above, would provide a single source for both individual infection risk and travel characteristics of persons enumerated. With HPM data being sparse and uncertain, alternative data sources, such as temporal sequences of satellite imagery of night time lights, may be used to assess the changing population densities of settlements through variations in illumination from fires and electric lighting, where large-scale seasonal migrations occur and compliment other HPM studies at a settlement resolution [101,102]. Finally, as receptivity is critical for assessing the patterns of onward transmission instigated by imported infections, compiling historical *Pf*PR data of relevance for receptivity mapping would aid future predictions of outbreaks and control needs, providing that factors such as urbanization and land use change that can permanently alter receptivity are accounted for. Projects such as The Human Mobility Mapping project [103] aims to provide open access to HPM databases and modelling frameworks through which malaria-relevant movement parameters can be quantified.

## Conclusion

Detailed spatial and temporal information on HPM can inform the strategic development of malaria control and elimination interventions, which if based according to geographical boundaries within which large infection flows occur, impact would be maximised. Cross-border initiatives between countries linked by significant HPM from high to low transmission areas are more likely to succeed in both achieving and maintaining elimination than single country strategies, which as shown previously, would face challenges through imported infections [104,105]. Therefore, identifying and quantifying HPM between and within countries is key for assessing elimination feasibility and useful for constructing effective control and elimination intervention strategies.

## Competing interests

The authors declare that they have no competing interests.

## Authors’ contributions

DKP did the literature search, identified datasets, carried out the analysis and wrote the first draft of the manuscript. AJG contributed to the analysis and review of the manuscript. AW contributed to the review of the manuscript. DLS contributed to the writing, analysis and review of the manuscript. COB contributed to the review of the manuscript. AMN contributed to the review of the manuscript. RWS contributed to the writing and review of the manuscript. AJT contributed to the writing, analysis and review of the manuscript. All authors read and approved the final version of the manuscript.
